# Supplementary material for: Increasing heart vascularisation after myocardial infarction using brain natriuretic peptide stimulation of endothelial and WT1+ epicardial cells
Source: eLife. 2020 Nov 27;9:e61050. doi: 10.7554/eLife.61050 (PMC7695454; doi:10.7554/eLife.61050)
Supplement: Supplementary file 1. [file elife-61050-supp1.docx]

**Supplemental File 1.** Antibodies used in flow cytometry analysis, immunohistology and Western blot analysis.

| Antibodies | Species | Dilution | Reference | usage |
| --- | --- | --- | --- | --- |
| **anti-goat Alexa 488** | chicken | 1/500 | Molecular Probes A21467 | Flow cytometry |
| **anti-rabbit Alexa 488** | donkey | 1/1000 | Molecular Probes A21206 | Flow cytometry |
| **CD31 Biotin** | rat | 1/50 | BD Biosciences 553371 | Flow cytometry |
| **GFP** | rabbit | 1/500 | Abcam ab290 | Flow cytometry |
| **Isotype control for CD31** | rat | 1/10 | BD Biosciences 553928 | Flow cytometry |
| **NPRA** | rabbit | 1/50 | Abcam ab70848 | Flow cytometry |
| **NPRB** | goat | 1/50 | Santa Cruz Sc-34421 | Flow cytometry |
| **Streptavidine APC** | rat | 1/1000 | BioLegend 405207 | Flow cytometry |
| **anti-goat Alexa 594** | donkey | 1/1000 | Molecular Probes A11058 | immunohistology |
| **anti-goat biotin** | horse | 1/200 | Vector BA-5000 | immunohistology |
| **anti-rabbit Alexa 488** | goat | 1/1000 | Molecular Probe A11034 | immunohistology |
| **anti-rabbit Alexa 488** | donkey | 1/1000 | Molecular Probes A21206 | immunohistology |
| **anti-rabbit Alexa 594** | donkey | 1/1000 | Molecular Probes A21207 | immunohistology |
| **anti-rat Alexa 488** | donkey | 1/1000 | Molecular Probes A21208 | immunohistology |
| **anti-rat Alexa 647** | donkey | 1/1000 | Jackson Immuno 712-605-150 | immunohistology |
| **BrdU** | rat | 1/100 | Abcam ab6326 | immunohistology |
| **CD31** | rabbit | 1/100 | Abcam ab28364 | immunohistology |
| **CD31 biotin** | rat | 1/100 | BD Biosciences 553371 | immunohistology |
| **CD45** | rabbit | 1/50 | Abcam ab10558 | immunohistology |
| **ckit** | rat | 1/100 | R&D Sytems MAB1356 | immunohistology |
| **GFP** | goat | 1/2000 | Abcam ab5450 | immunohistology |
| **NPRA** | rat | 1/50 | R&D Sytems MAB3974 | immunohistology |
| **NPRA** | rabbit | 1/50 | Abcam ab70848 | immunohistology |
| **NPRB** | goat | 1/50 | Santa Cruz Sc-34421 | immunohistology |
| **NPRB** | rabbit | 1/20 | Abcam ab139188 | immunohistology |
| **Sca-1** | rat | 1/1000 | Abcam ab51317 | immunohistology |
| **Streptavidine Alexa 594** |  | 1/1000 | Molecular Probe S11227 | immunohistology |
| **Streptavidine Alexa 647** |  | 1/1000 | Molecular Probe S32357 | immunohistology |
| **WT-1** | rabbit | 1/100 | Abcam ab89901 | immunohistology |
| **anti-mouse IRDye 800** | goat | 1/10000 | Rockland 610-132-121 | Western Blot |
| **anti-rabbit Alexa 680** | goat | 1/5000 | Molecular Probe A21109 | Western Blot |
| **CD31** | rabbit | 1/500 | Abcam ab28364 | Western Blot |
| **Phospholamban**  **Phospho phospholamban**  **Phospho p38**  **p38**  **tubulin** | rabbit  mouse  rabbit  rabbit  mouse | 1/1000  1/500  1/500  1/1000  1/10000 | Millipore  Millipore  Cell Signaling  Cell Signaling  Sigma T5168 | Western blot  Western blot  Western Blot/  immunohistology  Western blot  Western Blot |
|  |  |  |  |  |
