# Supplementary material for: Increasing heart vascularisation after myocardial infarction using brain natriuretic peptide stimulation of endothelial and WT1+ epicardial cells
Source: eLife. 2020 Nov 27;9:e61050. doi: 10.7554/eLife.61050 (PMC7695454; doi:10.7554/eLife.61050)
Supplement: Supplementary file 2. [file elife-61050-supp2.docx]

**Supplemental File 2:** Primer Sequences used in quantitative RT-PCR.

| Gene | Sense | Anti-sense | Product size (bp) |
| --- | --- | --- | --- |
| **18S** | ACTTTTGGGGCCTTCGTGTC | GCCCAGAGACTCATTTCTTCTTG | 96 |
| **acta2** | CAGGCATGGATGGCATCAATCAC | ACTCTAGCTGTGAAGTCAGTGTCG | 154 |
| **pecam1** | GCCTCACCAAGAGAACGGAAGGC | CTGCTTTCGGTGGGGACAGGC | 158 |
| **cd34** | CTTCTGCTCCGAGTGCCATT | GCCAAGACCATCAGCAAACAC | 250 |
| **kit** | ATCTGCTCTGCGTCCTGTTG | CTGATTGTGCTGGATGGATG | 108 |
| **nos3** | GGCTGTGGTAGTTAGGGCATC | AGGTTTGGGTTGGGCATCT | 165 |
| **kdr** | ACTGCAGTGATTGCCATGTTCT | CCTTCATTGGCCCGCTTAA | 74 |
| **Ly6a** | TTTGAGACTTCTTGCCCATC | ACCCAGGATCTCCATACTTTC | 159 |
| **Cdh5** | AGCGCAGCATCGGGTACT | TCGGAAGAATTGGCCTCTGT | 56 |
| **vegfa** | GTACCTCCACCATGCCAAGT | GCATTCACATCTGCTGTGCT | 340 |
| **vwf** | GATGCCCCAGTCAGCTCTAC | TCAGCCTCGGACAACATAGA | 131 |
| **wt1** | CACGGCACAGGGTATGAGAG | GTTGGGGCCACTCCAGATAC | 128 |
|  |  |  |  |
